# A simple and efficient *Agrobacteriumtumefaciens*-mediated plant transformation of *Brassica rapa* ssp. *pekinensis*

**DOI:** 10.1007/s13205-016-0402-1

**Published:** 2016-03-07

**Authors:** Venkidasamy Baskar, Baniekal H. Gangadhar, Se Won Park, Shivraj Hariram Nile

**Affiliations:** Department of Bioresources and Food Science, School of Life and Environmental Sciences, Konkuk University, Seoul, 143701 South Korea

**Keywords:** *Agrobacteriumtumefaciens*, Acetosyringone, *Brassica rapa*, Cultivation, Transformation

## Abstract

The present study aims to investigate the numerous factors influencing *Agrobacterium tumefaciens*-mediated genetic transformation of Chinese cabbage (*Brassica rapa* ssp. *pekinensis*). Factors affecting transformation efficiency, such as age of explants, *Agrobacterium* concentration, and effect of acetosyringone, pre-cultivation, infection and co-cultivation time of *Agrobacterium* were examined. The pre-cultured hypocotyls from young seedlings prior to exposure to *Agrobacterium* showed higher shoot regeneration. The plant transformation with the modest *A. tumefaciens* concentrations (0.8 OD) and the 3 days co-cultivation periods increased transformation efficiency. Plant growth hormones [1-naphthyl acetic acid (NAA) and 6-benzyl amino purine (BAP)] were essential for callus and shoot formation. Root formation was effective in half strength MS medium without supplementation of root-inducing hormones. To maintain selection pressure, plant subculture was carried out every 2 weeks with selective antibiotics. The putative transgenic plants were acclimatized in the greenhouse. Polymerase chain reaction was performed to confirm the integration of T-DNA into the genome of transgenic plants. A transformation efficiency of 15 % was obtained. This protocol allows effective transformation and indirect regeneration of *Brassica rapa*.

## Introduction

Several health-promoting compounds (phenolics, flavanoids) are rich in *Brassicaceae* plants (Jahangir et al. 2009). Moreover, glucosinolates (GSLs) are the major secondary metabolites found in the *Brassicaceae* family (Fahey et al. 2001). Glucosinolates provided resistance against pathogens and control auxin homeostasis in plants (Baskar et al. 2012). Among different Brassica crops, *Brassica rapa* ssp. *pekinensis* is globally cultivated and is highly consumed in Korea with the cultivation area covers across 37,200 ha (Park and Cheigh 2003). These natural health-promoting compounds should be enriched via the genetic engineering approaches for the potential use of *B. rapa* crops. However, the generation of transgenic plants in *B. rapa* is considered as difficult. Previous reports indicated the successful regeneration efficiencies in *B. rapa* for various purposes (Tang et al. 2003; Min et al. 2007; Zang et al. 2008). Zang et al. (2008) attempted to generate *B. rapa* transgenic plants to enrich glucosinolates. Similarly, Cho et al. (2001) successfully generated *B. rapa* transgenic plants expressing *CRY*-*1C* gene. However, the rate of transformation efficiency is largely varied. The effective in vitro plant regeneration system is essential for genetic transformation in plants. Here, we have established an efficient reproducible protocol for high-frequency regeneration via indirect organogenesis from hypocotyl explants of Chinese cabbage. In this paper, we described a simple protocol for *Agrobacterium*-mediated genetic transformation via indirect regeneration using hypocotyl explants in *B. rapa*.

## Materials and methods

### Surface seed sterilization

Seeds of *B. rapa*. ssp. *pekinensis* (Kyoungshin seed, Korea) variety Seoul was surface sterilized by immersing the seeds in 70 % ethanol for 1 min, and washed twice with sterile distilled water. Then seeds were soaked in 2 % sodium hypochlorite and stirred for 20 min followed by briefly rinsed with sterile deionised (DI) water. The surface-sterilized seeds were inoculated into Murashige and Skoog (Duchefa, Netherlands) medium solidified with 0.8 g l^−1^ phytoagar (Duchefa) and incubated at 21 °C, under a 16/8 h photoperiod.

### Agrobacterium-mediated plant transformation

**Fig. 1:**
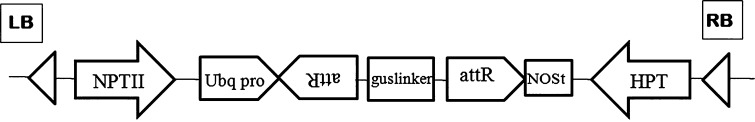
Schematic representation of the T-DNA region of pANDA vector. *LB* left border, *RB* right border, *NPTII* neomycin phosphotransferase II, *Ubq pro* ubiquitin promoter, *attR* att sites, *NOSt* nopaline synthase terminator, *HPT* hygromycin phosphotransferase, *RB* right border

Hypocotyls of 7–10 mm excised from the 5-day-old seedlings of *B. rapa* were pre-cultured for 3 days at 21 °C, under a 16/8 h photoperiod in MS medium with 0.8 % phytoagar + 5 mg l^−1^ acetosyringone + 4 mg l^−1^ 6-benzyl amino purine (BAP) + 3 mg l^−1^ 1-naphthyl acetic acid (NAA) + 4 mg l^−1^ AgNO_3_). The plant expression vector (pANDA) carrying *A. tumefaciens* EHA105 strain was streaked on yeast extract peptone agar (YEP) medium with hygromycin (50 mg l^−1^) and rifampicin (25 mg l^−1^) antibiotics and incubated at 28 °C for 2 days. The graphical representation of T-DNA region of pANDA vector is shown in Fig. 1. The fresh culture was inoculated into YEP broth medium supplemented with respective antibiotics and 5.0 mg l^−1^ acetosyringone and incubated at 28 °C for overnight. The bacterial growth was measured in UV–visible spectrophotometer (UV-2550, Shimadzu) using optical density at 600 nm and the 0.8 OD density culture was used for plant transformation. The overnight-grown bacterial culture was centrifuged at 3500 rpm for 10 min. The bacterial pellet was resuspended in the infection medium (Liquid MS, pH 5.2 + 5.0 mg l^−1^ acetosyringone). The pre-cultured hypocotyl explants were incubated with *Agrobacterium* suspended in infection medium for 30 min and then transferred to the sterile autoclaved blot paper to remove the excess bacterial strains. *Agrobacterium*-infected hypocotyls were co-cultivated on MS medium with 0.8 % phytoagar + 5.0 mg l^−1^ acetosyringone + 4.0 mg l^−1^ BAP + 3.0 mg l^−1^ NAA + 4.0 mg l^−1^ AgNO_3_ and incubated in the dark at 21 °C for 3 days; followed by which the hypocotyls were transferred to the MS medium supplemented with 1.6 % phytoagar + 4.0 mg l^−1^BAP + 3.0 mg l^−1^ NAA + 4.0 mg l^−1^ AgNO_3_ + 250 mg l^−1^carbenicillin + 10 mg l^−1^ hygromycin to induce callus formation. The explants were subcultured in the same medium for every 15 days. Selected green callus was transferred to shoot induction medium (MS medium with 1.6 % phytoagar + 4.0 mg l^−1^ BAP + 0.5 mg l^−1^ NAA + 4.0 mg l^−1^ AgNO_3_ + 250 mg l^−1^ carbenicillin + 10 mg l^−1^ hygromycin). After 2 months, the selected shoots were transferred to rooting medium (half strength MS + 0.8 % phytoagar + 250 mg l^−1^ carbenicillin + 10 mg l^−1^ hygromycin).

### Confirmation of transgenic plants using PCR

Genomic DNA was isolated from the leaves of putative rooted transgenic Chinese cabbage plants using DNA extraction kit (DNeasy Plant Mini Kit, Qiagen, USA). PCR was performed using the gus linker primers F: 5′-CATGAAGATGCGGACTTACG-3′ and R: 5′-ATCCACGCCGTATTCGG-3′. The PCR conditions were performed as follows: 95 °C for 5 min, 30 cycles of 95 °C for 1 min, annealing temperature for 1 min, extension at 72 °C for 1 min and final extension at 72 °C for 7 min. The PCR products were run in 1 % agarose gel electrophoresis and viewed under UV transilluminator.

### Statistical analysis

All experiments were carried out three times with three replicates. Statistical analysis was performed using one-way analysis of variance (ANOVA) followed by Duncan’s test to determine significant (*P* ≤ 0.05) differences. The standard error was calculated using the n values of each experiment (*n* = 3).

### Results and discussion

Chinese cabbage (*B. rapa* ssp. *pekinensis*) has been cultivated globally and consumed in various regions in the world. It has rich phytochemical (glucosinolates, phenolics, flavanoid and carotenoids) composition. The efficient plant transformation method is needed to improve the nutritive values of the plants. Previous study has reported the recalcitrant property of *B. rapa* to *Agrobacterium*-mediated transformation (Liu et al. 2012). *Agrobacterium* plant transformation methods were commonly used for the production of transgenic Chinese cabbage (*B. rapa* ssp. *pekinensis*) (Tang et al. 2003; Min et al. 2007; Zang et al. 2008). Here, we provided the various factors influencing *Agrobacterium*-mediated plant transformation in *B. rapa* ssp. *pekinensis* and the transgenic plants were confirmed using PCR.

### Organogenesis from hypocotyl explants

Different plant growth regulators can directly influence callus induction, shoot bud formation, shoot elongation and rooting from cotyledonary and hypocotyl explants of *B. rapa*. MS medium supplemented with BAP (4.0 mg l^−1^) and NAA (1.0 mg l^−1^) were used in *B. rapa* for shoot regeneration in earlier studies (Min et al. 2007). Similarly, in the present study, MS medium supplemented with BAP (4.0 mg l^−1^) and NAA (3.0 mg l^−1^) is considered as suitable for callus induction and BAP (4.0 mg l^−1^) and NAA (0.5 mg l^−1^) have been selected for shoot induction. This indicated that BAP and NAA were sufficient for the callus induction and shoot bud regeneration. The calluses induced in the compact were green and showed higher shoot regeneration frequency (15 shoots). The elongated shoots were rooted (78.0 %) in half strength MS medium with the absence of plant hormones (Table 1). According to previous reports, we did not find the differences in rooting between MS medium and MS medium supplemented with rooting hormones (NAA, IAA) (Vanjildorj et al. 2009; Lee et al. 2004). Rooted plants were acclimatized in the greenhouse (85 %).

**Table 1:** Effect of growth regulators on callus induction, shoot formation, and root induction of *Brassica rapa* ssp. *pekinensis*

Growth regulators (mg l^−1^)	Callus per hypocotyl explants (%)	No. shoots from hypocotyl calli	Root induction (%)
BAP + NAA + AgNO_3_			
1.0 + 3.0 + 4.0	35.25 ± 2.10^d^		
2.0 + 3.0 + 4.0	49.00 ± 2.76 ^cd^		
3.0 + 3.0 + 4.0	65.00 ± 1.15^b^		
4.0 + 3.0 + 4.0	85.51 ± 1.12^a^		
5.0 + 3.0 + 4.0	51.42 ± 2.00^c^		
BAP + NAA + AgNO_3_			
4.0 + 3.0 + 4.0		1.52 ± 0.50^e^	
4.0 + 2.0 + 4.0		5.55 ± 0.75^d^	
4.0 + 1.0 + 4.0		9.21 ± 0.75^c^	
4.0 + 0.5 + 4.0		15.00 ± 0.55^a^	
MS full strength			59.00 ± 1.15^b^
MS half strength			78.00 ± 1.00^a^
MS + IAA 1.0			49.52 ± 0.95^c^
MS + IBA 1.0			41.23 ± 1.01^d^

Data represent mean values ± standard error (SE) of three replicates; each experiment was repeated thrice. Means with common letters are not significantly different at *P* ≤ 0.05 according to Duncan’s multiple range test (DMRT)

### Influence of antibiotics on shoot regeneration from hypocotyl explants

To determine the appropriate concentration of selection agent to effectively screen transformed shoots, we cultured hypocotyl explants on callus induction medium (CIM) supplemented with different concentrations of hygromycin. After 3 weeks of culture, 74.51 % callus induction was attained in explants cultured on CIM lacking hygromycin. At 5.0 mg l^−1^, 90 % of explants bleached and died. Further increase in the level of hygromycin to 10 and 15 mg l^−1^ totally inhibited callus induction. To minimize escape and prevent necrosis, we have selected 10 mg l^−1^ hygromycin for the transformation experiments. The present investigation correlates with the previous studies conducted by Vanjildorj et al. (2009) in which 10 mg l^−1^ hygromycin was used for the selection of transgenic plants in *B. rapa* ssp. *pekinensis*. Carbenicillin (250 mg l^−1^) was used to kill *Agrobacterium* after co-cultivation with explants. The obtained results were paralleled with the earlier study reported by Vanjildorj et al. (2009) in which the authors used the same concentration as optimum for removing *Agrobacterial* growth in *B. rapa* ssp. *pekinensis.*

### Evaluation of factors influencing transformation

Plant transformation was standardized with preliminary experiments. In the present study, we found that 3-day-pre-cultured hypocotyls excised from 5-day-old seedlings showed good response which is similar to the previous study that indicated the use of 4-day-old cotyledonary explants of *B. rapa* showed higher regeneration (Vanjildorj et al. 2009). Among the different age of explants, 5-day-old seedlings show higher regeneration. Pre-culture of explants is a critical factor to achieve high frequency of transformation. It makes the tissues competent enough to withstand the bacterial infection and increased the production of gene integration. Similarly, pre-culturing of explants prior to infection with *Agrobacterium* enhanced the transformation frequency (Min et al. 2007). Bacterial cell density as measured by the optical density of bacterial suspension is directly related to their cell mass or cell number. Selection of appropriate OD is an important factor of concern for *Agrobacterium* genetic transformation. Lower densities (0.2 and 0.6) were not effective for transformation, whereas the highest density (1.0) decreased infection efficiency (Table 2). Kong et al. (2009) demonstrated that *Agrobacterium* cell density of 0.8 OD induced higher frequency of transformation in *B. napus*. Overgrowth of *Agrobacterium* was the real problem, when infection time increased to 45 min. Similarly, increase of infection time to more than 35 min caused browning of the target tissue and did not allow flourishing. Therefore, optimized OD (0.8) was used with 30 min of infection time in further experiments. Table 2 shows that addition of 5.0 mg l^−1^ acetosyringone to *Agrobacterium* culture had significant improvement of the transformation frequency (15.53 %) compared to the *Agrobacterium* culture without acetosyringone. Consistent with our results, several fold increase in transformation efficiency has been noted when 200 µM acetosyringone was used in broccoli transformation (Henzi et al. 2000). To determine the most suitable duration of co-cultivation, explants were co-cultivated with *A. tumefaciens* for 0–4 days in the dark. In the present study, we found that the combination of 3-day pre-cultivation and 3-day co-cultivation period increased the transformation frequency. Co-cultivation in dark condition increased the transformation frequency in *B. napus* (Kong et al. 2009). Further, the majority of the explants perished due to bacterial overgrowth when the co-cultivation period exceeded 3 days. In agreement with this report, we also found that plants co-cultivated for 3 days in the dark act as optimum for *Agrobacterium* transformation.

**Table 2:** Factors affecting transformation frequency of *Brassica rapa* ssp. *pekinensis*

Factors	No. of explants cultured	Transformation frequency (%)
Pre-culture days		
0	30	0.0
1	30	7.0 ± 1.10^e^
2	30	11.5 ± 2.00^bc^
**3**	30	**15.5** **±** **1.00** ^**a**^
4	30	10.5 ± 1.10^d^
OD value		
0.2	30	0.0
0.4	30	6.5 ± 1.00^ef^
0.6	30	10.5 ± 1.50^d^
**0.8**	30	**15.5** **±** **0.72** ^**a**^
1.0	30	11.0 ± 1.25^c^
Co-culture period		
0	30	0.0
1	30	6.0 ± 1.00^f^
2	30	12.2 ± 1.00^b^
**3**	30	**15.5** **±** **1.12** ^**a**^
4	30	10.0 ± 1.20^d^
Acetosyringone (mg l^−1^)		
0	30	0.0
2	30	2.0 ± 1.00^f^
4	30	12.2 ± 1.00^b^
**5**	30	**15.5** **±** **0.50** ^**a**^
**6**	30	10.0 ± 1.20^d^

The best combinations of different factors (pre-culture, OD value, co-culture & acetosyringone) are shown in *block* *letters*. Data represent mean values ± standard error (SE) of three replicates; each experiment was repeated thrice. Means with common letters are not significantly different at *P* ≤ 0.05 according to Duncan’s multiple range test (DMRT)

### Genetic transformation of hypocotyl explants

**Fig. 2:**
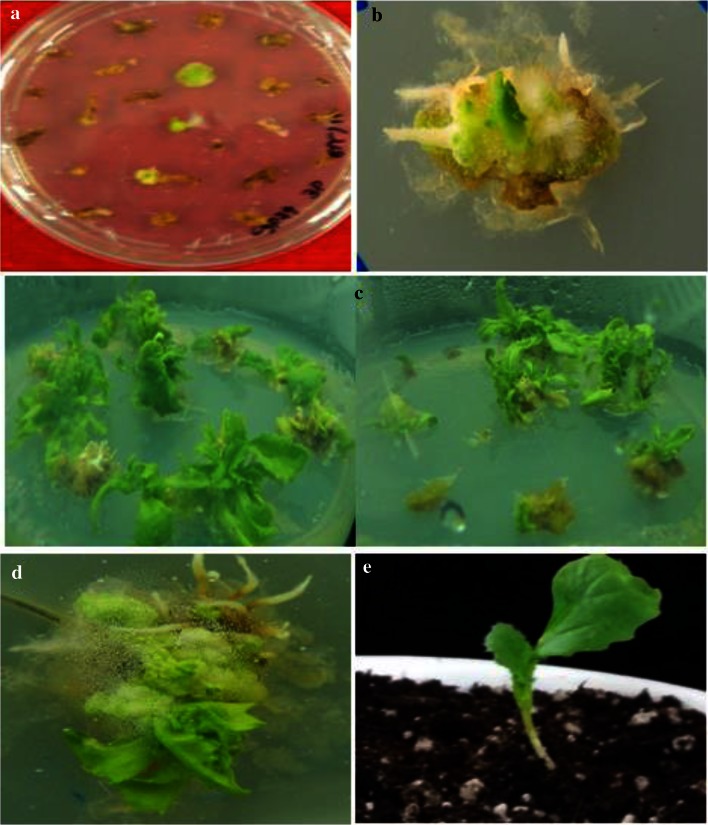
Figure showing the formation of **a** callus induction **b** shoot induction **c** multiple shoot formation **d** root induction **e** hardening plants from the hypocotyls of *Agrobacterium*-transformed *Brassica rapa* ssp. *pekinensis* plants

Callus initiation was observed in selective medium (MS medium containing BAP (4.0 mg l^−1^), NAA (3.0 mg l^−1^), AgNO_3_ (4.0 mg l^−1^) carbenicillin (250 mg l^−1^) and hygromycin (10 mg l^−1^) within 2 weeks of culture (Fig. 2). The untransformed control explants on selective medium turned yellow and did not produce calli. Putative transgenic callus were transferred to MS medium containing 4.0 mg l^−1^ BAP, 0.5 mg l^−1^ NAA, 4.0 mg l^−1^ AgNO_3_, 10 mg l^−1^ hygromycin and 250 mg l^−1^carbenicillin for shoot bud initiation within 2 weeks (Fig. 2). Shoots that survived this selection stage were transgenic and were transferred to rooting medium (half strength MS medium and 10 mg l^−1^ hygromycin in the absence of plant hormones) for 3 weeks. The rooted plantlets were transferred to pots, acclimated for 2 weeks in the culture room and were moved to the greenhouse.

### Molecular confirmation of transgenic plants

**Fig. 3:**
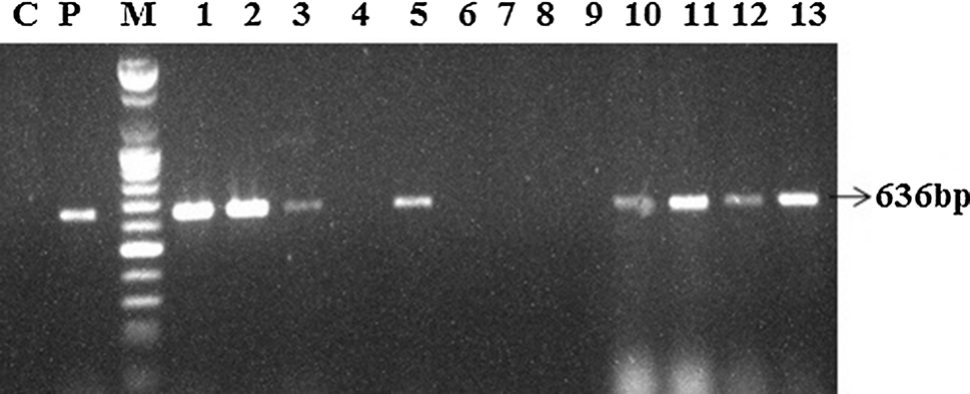
Genomic DNA PCR amplification of gus linker sequences from the representative transgenic *Brassica rapa* ssp. *pekinensis* plants. *C* control plant, *P* positive control, *M* 100 bp ladder, *1–12* putative transformed plants

The putative transgenic rooted plants were subjected to PCR analysis. PCR was carried out using the DNA extracted from the leaves of putative transgenic plants (Fig. 3). PCR analysis showed the amplification of the predicted 636 bp fragments corresponding to the gus linker gene whereas untransformed plants did not show PCR amplifications. In summary, we described the factors influencing *Agrobacterium*-mediated plant transformation in *B. rapa* ssp. *pekinensis*. We found that the age of explants and medium conditions influenced the regeneration. In our study, age of explants, effect of acetosyringone, *Agrobacterium* concentration, pre-cultivation, and co-cultivation time significantly increased the transformation frequency of *B. rapa* ssp. *pekinensis*.

## Abbreviations

BAP: N6-benzyl amino purine
IAA: Indole-3-acetic acid
IBA: Indole-3-butyric acid
MS: Murashige and Skoog
NAA: Naphthalene acetic acid
